# Physical activity, sleep and cardiovascular health data for 50,000 individuals from the MyHeart Counts Study

**DOI:** 10.1038/s41597-019-0016-7

**Published:** 2019-04-11

**Authors:** Steven G. Hershman, Brian M. Bot, Anna Shcherbina, Megan Doerr, Yasbanoo Moayedi, Aleksandra Pavlovic, Daryl Waggott, Mildred K. Cho, Mary E. Rosenberger, William L. Haskell, Jonathan Myers, Mary Ann Champagne, Emmanuel Mignot, Dario Salvi, Martin Landray, Lionel Tarassenko, Robert A. Harrington, Alan C. Yeung, Michael V. McConnell, Euan A. Ashley

**Affiliations:** 1 Department of Medicine, Stanford University, Stanford, California USA; 2 Division of Cardiovascular Medicine, Department of Medicine, Stanford University, Stanford, California USA; 3 Sage Bionetworks, Seattle, WA USA; 4 Ted Rogers Centre of Excellence for Heart Function, Toronto, Ontario Canada; 5 Stanford Center for Cardiovascular Innovation, Stanford University, Stanford, California USA; 6 Stanford Center for Biomedical Ethics, Stanford University, Stanford, California USA; 7 Stanford Center on Longevity, Stanford University, Stanford, California USA; 8 Stanford Prevention Research Center, Stanford University, Stanford, California USA; 9 Stanford Sleep Center, Stanford University, Palo Alto, California USA; 10 Institute of Biomedical Engineering, Department of Engineering Science, University of Oxford, Oxford, United Kingdom; 11 Big Data Institute, Nuffield Department of Population Health, University of Oxford, Oxford, United Kingdom; 12 Verily Life Sciences LLC, South San Francisco, California USA; 13 Department of Genetics, Stanford University, Stanford, California USA; 14 VA Palo Alto Health Care System, Palo Alto, California USA

**Keywords:** Data acquisition, Risk factors

## Abstract

Studies have established the importance of physical activity and fitness for long-term cardiovascular health, yet limited data exist on the association between objective, real-world large-scale physical activity patterns, fitness, sleep, and cardiovascular health primarily due to difficulties in collecting such datasets. We present data from the *MyHeart Counts* Cardiovascular Health Study, wherein participants contributed data via an iPhone application built using Apple’s ResearchKit framework and consented to make this data available freely for further research applications. In this smartphone-based study of cardiovascular health, participants recorded daily physical activity, completed health questionnaires, and performed a 6-minute walk fitness test. Data from English-speaking participants aged 18 years or older with a US-registered iPhone who agreed to share their data broadly and who enrolled between the study’s launch and the time of the data freeze for this data release (March 10 2015–October 28 2015) are now available for further research. It is anticipated that releasing this large-scale collection of real-world physical activity, fitness, sleep, and cardiovascular health data will enable the research community to work collaboratively towards improving our understanding of the relationship between cardiovascular indicators, lifestyle, and overall health, as well as inform mobile health research best practices.

## Abstract (summary)

**Table:** 

Design Type(s)	observation design · source-based data analysis objective · data collection and processing objective
Measurement Type(s)	physical activity · sleep
Technology Type(s)	crowd-sourced data generation
Factor Type(s)	sex · height · weight · age · smoking status measurement · employment status
Sample Characteristic(s)	Homo sapiens · United States of America

Machine-accessible metadata file describing the reported data (ISA-Tab format)

## Background and Summary

Mobile technology, in particular advances in smartphone sensors, offers an opportunity to evaluate and monitor cardiovascular health and fitness^1,2^ with unprecedented connectivity. Direct measurement of activity through “always-on”, low-power motion chips allows for objective, real-world measurements of physiologic parameters. The widespread use of smartphones globally could thus transform research in this area and potentially improve clinical outcomes^3–7^.

In March 2015, *MyHeart Counts* (https://github.com/ResearchKit/MyHeartCounts) was launched as an observational smartphone-based study developed using Apple’s ResearchKit software development library (http://researchkit.org/). The study’s goal is to evaluate the feasibility of frequent, remote data sampling of physiologic parameters as measured by smartphone measures of fitness, activity, and sleep. These data may facilitate a more complete understanding of the association between objective measures of health, self-reported disease, and quality of life. Researchers may use these data to characterize activity profiles as to better understand the impact of the number of activity transitions on health^8^. There are many questions that may stem from these data, each of which will require a community of researchers to explore.

Findings from the MyHeart Counts study revealed a clustering of participants by activity pattern into several distinct groups–sedentary, active, active only on workdays, active only on non-workdays. These cluster assignments were found to correlate with participants’ self-reported incidence of cardiovascular disease as well as self-reported mental well-being. Additionally, the MyHeart Counts study results suggest that patterns of activity correlate with different incidence of self-reported cardiovascular disease: individuals with multiple short bursts of physical activity throughout the day reported better health as compared to counterparts who performed the same number of minutes of physical activity, but in one longer session. These findings are described in *JAMA Cardiology*^8^.

**Fig. 1:**
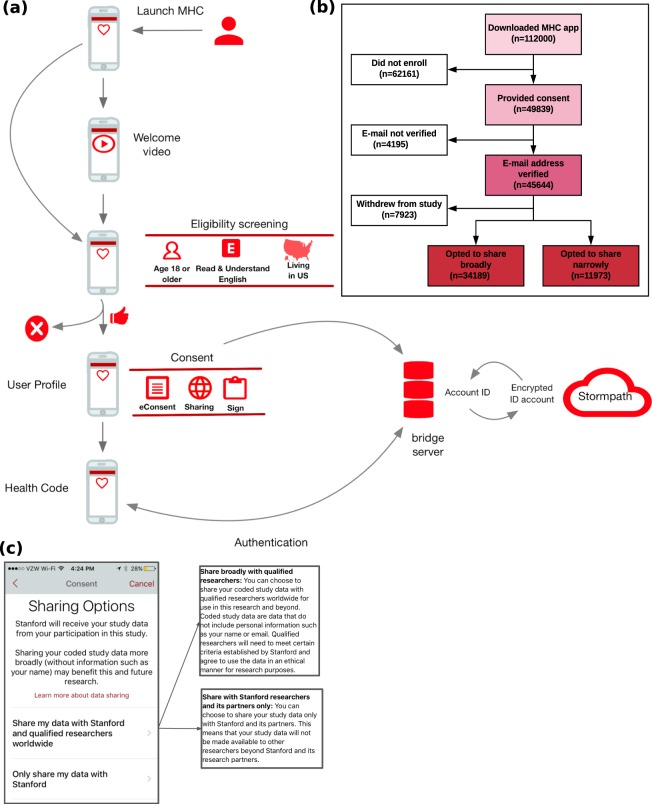
Onboarding, flow, and demographics for the *MyHeart Counts* study. (**a**) Study design. (**b**) CONSORT diagram of participant flow through the study. (**c**) Screenshot of sharing options from the MyHeart Counts app.

*MyHeart Counts* utilized remote enrollment and consent in which participants self-guide through a visually engaging eConsent, in addition to a traditional consent form, prior to deciding to join the study^9^ (Fig. 1). A critical aspect of this transparent consent process is providing participants with an explicit decision point, allowing them to specify if the de-identified data they donate to the study can also be studied by qualified researchers worldwide^10^.

The MyHeart Counts iOS app was downloaded among 110,056 users from March 9, 2015 to October 28, 2015. The number of users who enrolled in the study, consented and shared data worldwide (broadly) and Stanford only (narrow) is shown in Fig. 1. The study cohort described here is composed of contributions from study participants who designated broad sharing of their data (n = 34,189).

Among consented participants, 4,900 (10.2%) completed a 6-minute walk test at the end of day 7 in the study. The 6-minute walk test activity not only collects distance traveled, but also accelerometry during the walk. This comprises the largest 6-minute walk data cohort to date^11–15^. Additionally, *MyHeart Counts* users were able to upload data from wearable devices compatible with HealthKit. The most popular wearable devices used are listed in Table 1. These additional data from wearables enabled more continuous monitoring of activity patterns than through the phone alone.

**Table 1:** Top five external applications (ranked by number of unique users) that provided data to HealthKit Data, Workout, and Sleep collectors.

HealthKit Data			HealthKit Workout			HealthKit Sleep		
Application	#Ind.	# Per.-Days.	Application	# Ind.	#Per.-Days	Application	# Ind.	#Per.-Days
MyFitnessPal	933	18,210	Strava	195	3,275	Apple Mobile Timer	636	14,523
Withings	607	30,699	Runkeeper	134	1,556	Lexwarelabs Sleep Cycle	439	31,872
Lose It!	387	5,289	Humanco	127	6,139	Tantsissa Autosleep	366	10,293
SleepHealth	286	294	Nikeplus Running	111	542	Sleep++	210	6,647
Strava	269	2,817	Garmin Connect	95	1,817	Neybox Pillow	184	3,088

Our aim in sharing data donated by *MyHeart Counts* participants is to encourage the consolidation of a broad, diverse, and collaborative community of mobile health researchers. We invite diverse solvers from around the world to engage in better understanding how mobile technologies can impact cardiovascular health.

## Methods

These methods are expanded versions of descriptions in our related work^8^.

### Participant onboarding

The *MyHeart Counts* app was made available starting in March 2015 through the Apple App Store (https://itunes.apple.com/us/app/myheart-counts/id972189947?mt=8) in the United States for iPhone 4S or newer requiring a minimum of iOS 8. Enrollment was open to individuals 18 years of age or older who were able to read and understand English and had iPhones registered in the United States. Participants then completed an interactive eConsent process that included animated icons, concise text, and links for more information^16^. In completing the eConsent, participants designated a data sharing preference: only with Stanford (“narrow sharing”) or more broadly with qualified researchers worldwide (no default choice was presented) (Fig. 1c).

After completing the eConsent, participants were asked to e-sign an electronically rendered traditional consent form. A copy of the signed consent document was sent to participants by email, allowing for verification of their enrollment in the study. Following enrollment, participants could choose their next actions within the study, including setting a 4 digit passcode or registering a fingerprint scan to secure the study app, or completing preliminary study activities. These data were sent to Stormpath, a service used by the bridge server to perform login and store PHI separately from other forms of study data. As part of onboarding, participants were invited to grant the study app access to their iPhone’s HealthKit, Motion Activity, Notifications, and Location Service. Ethical oversight of the study was obtained from Stanford University’s Research Compliance Office (Protocol #IRB-31409).

### Study tasks

Consented participants contributed a range of data passively, as well as data that were contributed actively through forms and surveys, and via the 6-minute walk test.

Data collected as part of onboarding included participant account information (name, email, password), as well as study data (gender, height, weight, wake & sleep times). These study data were sent to the server the first time the participant opened the study application after verifying their email post-consent.

We enabled passive data collection from HealthKit and Core Motion when the participant opened their study app for the first time after verifying their email. HealthKit is a framework designed to capture, store, and facilitate sharing of health and physical activity data collected from iPhone sensors between apps. Additionally, a variety of apps and devices may write to HealthKit (e.g., Fitbit Sync Helper, Nike+ Run Club, Apple Watch, Beddit). The *MyHeart Counts* app captures a variety of body measurements (height, weight), physical activity data (active energy expenditure in kcal, cycling distance, flights climbed, sleep analysis, stand hours, steps, walking and running distance, workouts), health results (blood glucose), and vital signs (diastolic/systolic blood pressure, oxygen saturation) if they have been entered in HealthKit.

With users’ permission, during the initial 7-day monitoring period, motion was recorded through the Core Motion coprocessor chip of their iPhone (iPhone 5S or newer). The low-power chip integrates a number of sensor signals, including a triaxial accelerometer, gyroscope, compass, and barometer, to estimate the presence of movement, distance traveled, as well as the modality of movement, (i.e., walking, running, cycling, driving). Throughout the study, users were able to visualize these data on a dashboard built into the app. Data were sent to the server whenever 50 Kb was collected or when older than 24 h, using Wifi or cellular.

On the final day of the study (eighth day post-enrollment), participants were presented with a final set of questionnaires. These consisted of the Well-Being and Risk Perception Survey with additional questions used to compute the participant’s Atherosclerotic Cardiovascular Disease Risk Score^17,18^ from which a Heart Age^19^ was calculated. All survey questions as well as app screenshots of the survey presentation are available on the Synapse MyHeart Counts Public Researcher Portal (Wiki, Data Description, Survey Data Gathered in the MyHeart Counts App^20^.

During the same interval, participants were asked to complete a self-administered 6-minute walk test. The 6-minute walk test is a phone-guided task that triggers the collection of global positioning system displacement-based distances, pedometer-based distances, pedometer step counts, and accelerometer and gyroscope measurements in both raw and processed formats.

**Fig. 2:**
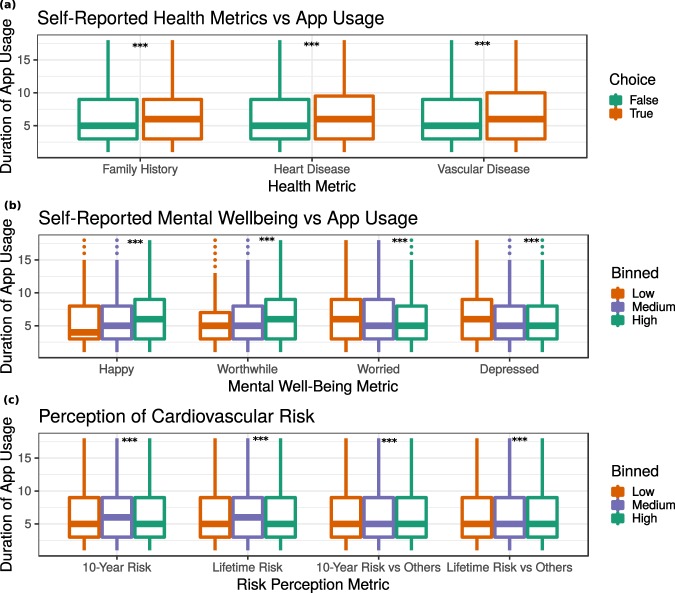
App engagement (as duration in days). (**a**) Self-reported cardiovascular health: family history of cardiovascular disease (padj = 1.73e-4, diff = 0.23+/−0.13); heart disease (pad = 8.78e-10, diff = 0.56+/−0.18); vascular disease (padj = 1.90e-4, diff = 0.47+/−0.25). (**b**) Self-reported mental wellbeing: feel worthwhile (pad < 2.20e-16, beta = 0.13+/−0.013); feel happy (padj < 2.2e-16, beta = 0.12+/−0.12); feel worried (padj < 2.2e-16, beta = −0.13+/−0.01); feel depressed (padj < 2.2e-16, beta = −0.094). (**c**) Perceived risk of cardiovascular disease: perceived 10-year risk (padj,2.2e-16, beta = 0.29+/−0.03); perceived lifetime risk (padj = 3.18e-5, beta = 0.105+/−0.02); perceived 10-year risk compared to others (p = 7.93e-4; beta = 0.079+/−0.02); perceived lifetime risk compared to others (padj = 2.81e-3, beta = 0.079+/−0.02). N = 45,656 participants.

Correlation analysis was performed to determine whether a participant’s duration of app usage during the first 8 days post-enrollment was associated with responses to the above-mentioned surveys (Fig. 2). It was found that participants with self-reported heart disease, vascular disease, and family history of heart disease used the app longer (Fig. 2a). Specifically, family history of heart disease correlated with 0.23+/−0.13 (p = 1.734e-4) more days of app usage, presence of heart disease correlated with 0.56+/−0.18 (p = 8.78e-10) more days of app usage, and presence of vascular disease correlated with 0.47+/−0.25 (p = 1.90e-4) more days of app usage. Similarly, participants’ mental well-being correlated with app usage (Fig. 2b). Participants who reported scores of 8–10 on the “feel worthwhile” and “happy” questions used the app longer as compared to those who reported low (1–3) or medium (4–7) values. Conversely, those who reported high values on the “worried” and “depressed” questions were found to use the app for a shorter period of time. Self-perceived risk was also significantly associated with duration of app usage (Fig. 2c). On the scale for this survey, those with a medium (score = 3) self-perceived 10-year risk used the app 0.29+/−0.03 (p < 2.2e-16) days longer than those with low (score = 1 or 2) or high (score = 4 or score = 5) perceived risk.

### Data collection and distribution

Data were sent by the app in encrypted form to Bridge Server, a RESTful API and researcher web interface developed and operated by Sage Bionetworks (http://sagebase.org/) and run on Amazon Web Services (AWS). Bridge is designed to allow collection and management of mobile health data from apps by providing apps the ability to securely create accounts for participants. The server then records consent and identifying personal information required for account creation separately from study data. Separation of personal information from study data is accomplished by storing personal information and accounts in a separate accounts database, and storing study data is S3 buckets on AWS. A dictionary stored in the Bridge server can convert an account identifier, used by the app when sending data, into a healthCode, used by the research team to identify an individual in the coded data (https://developer.sagebridge.org/articles/security.html).

Coded study data, consisting of survey responses, mobile sensor measurements and device data was exported to Synapse (https://www.synapse.org/) for distribution to researchers. Synapse^21^ is a general-purpose data and analysis sharing service where members can work collaboratively, analyze data, share insights, and track the attribution and provenance of those insights to share with others. Synapse is developed and operated by Sage Bionetworks as a service to the biomedical research community. These Bridge and Synapse services have been used to support numerous health studies, including all five of the initial ResearchKit apps launched in March 2015^8,22,23^ as well as subsequent studies^24^.

Multiple updates of the *MyHeart Counts* app were released during the study period to address software-related concerns and to implement new features. Because of an initial technical issue with the integration of HealthKit and ResearchKit data, demographic information is missing for a number of early participants. Participants were subsequently emailed to request they upgrade so this missing information could be provided.

## Data Records

Data was restricted to records shared by versions of the app released before October 28, 2015 (Version 1.0, 1.0.2, 1.0.3, 1.0.4, 1.0.5, 1.0.6, 1.0.7, 1.0.8, 1.5.0 [AKA 1.5.1 build 10]). For tables containing records lacking an AppVersion column, such as HealthKit and 6MWT Displacement data, data sent before October 28, 2015 was included. A total of 48,968 participants consented to the study and agreed to share their data broadly with the research community. 40,017 participants completed at least one survey or task after joining the study, of whom 34,189 agreed to share their data broadly. 6,870 completed all surveys presented in the first 8 days of their participation and were ages 40–70 years, allowing for computation of their 10-year risk score. 4990 completed at least one 6MWT with 6,927 total 6MWT completed. Clinical and demographic characteristics are provided in Table 2.

**Table 2:** Clinical and demographic characteristics of MHC users.

Characteristic	Count (n = 40,017)	%Dist
**Age**		
<30	6,351	30.62%
30–39	5,723	27.59%
40–49	3,696	17.82%
50–59	2,377	11.46%
60–69	1,769	8.53%
≥70	825	3.97%
NA	15,998	
**Gender**		
Female	4,952	22.39%
Male	17,151	77.55%
Other	12	<1%
NA	17,901	
Race or ethnic group	#	%
Alaska Native	5	<1%
American Indian	40	<1%
Asian	765	8.82%
Black	288	3.32%
Hispanic	631	7.27%
I prefer not to indicate	92	1.06%
Pacific Islander	27	<1%
White	6,606	76.15%
Other	220	2.53%
NA	31,343	
**Education level**		
Didn’t go to school	7	<1%
Grade school	152	1.97%
High school Diploma or GED	569	7.37%
Some college or vocational or associate	1,737	22.52%
College Bachelor’s	2,803	36.34%
Master’s Degree	1,601	20.75%
Doctoral Degree	844	10.94%
NA	32,304	
**Clinical History Smoking Status**		
TRUE	628	4.37%
FALSE	13,733	95.63%
NA	25,656	
Heart Disease*		
Present	3,185	14.18%
Absent	19,272	85.82%
NA	17,560	
Vascular Disease**		
Present	1,198	5.58%
Absent	20,269	94.42%
NA	18,550	
Family History of heart disease Father or brother with heart attack or coronary artery disease before age 55 y	3,890	18.00%
Mother or sister with heart attack or coronary artery disease before age 65 y	1,600	7.40%
None	16,144	74.60%
NA	18,383	
**Medications**		
To treat and lower cholesterol	2,904	12.40%
To treat hypertension and lower blood pressure	3,385	
To treat diabetes or prediabetes and lower blood glucose level	698	
None	16,364	
NA	16,666	

**Fig. 3:**
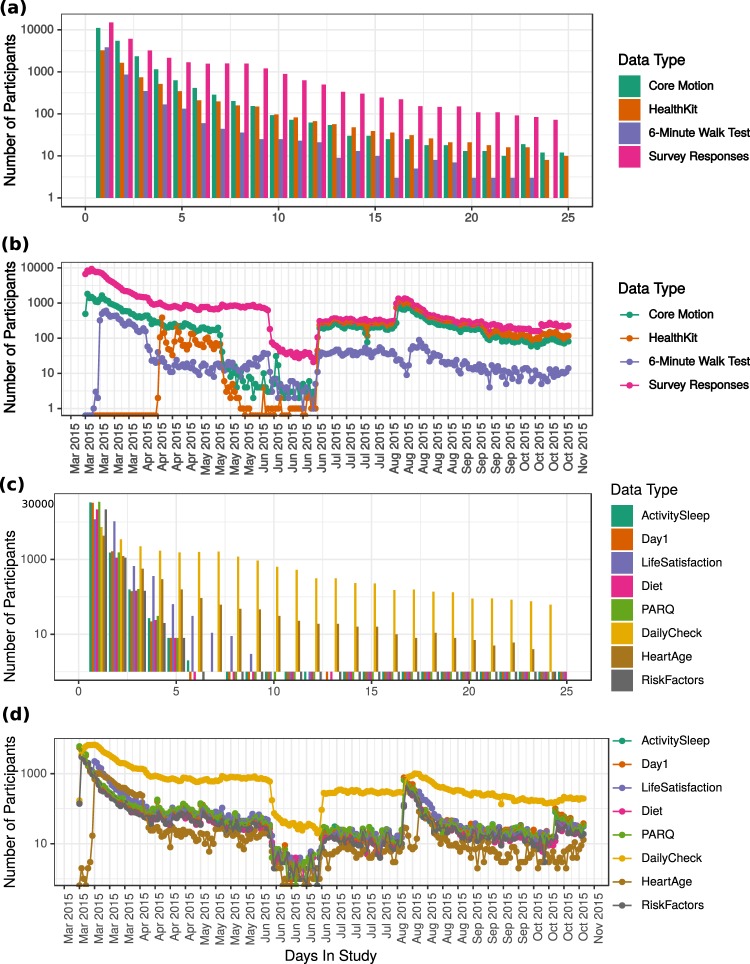
User engagement with the *MyHeart Counts* app from date of release (March 2015) to date of study completion (October 28, 2015). (**a**) Distribution of user participation by number of days they remained in the study. Number of days of core motion data is indicated in cyan; number of days of HealthKit data is indicated in orange; number of days with 6-Minute Walk Test data is indicated in purple; number of days with survey response is indicated in magenta. (**b**) Number of users who provided app data on each day during the study duration from March 2015 - October 2015. Core Motion data is indicated in cyan; HealthKit data is indicated in orange; 6-Minute Walk Test data is indicated in purple; survey responses are indicated in magenta. (**c**) Distribution of user participation by number of days they remained in the study. (**d**) Number of survey responses on each day during the study duration.

The number of study participants who provided daily HealthKit step data and activity pattern data derived from the phone’s core motion accelerometer are illustrated in Fig. 3. As an example, the distribution of the total number of days of motion and HealthKit step data provided by users during the study period are also illustrated in Fig. 3.

**Fig. 4:**
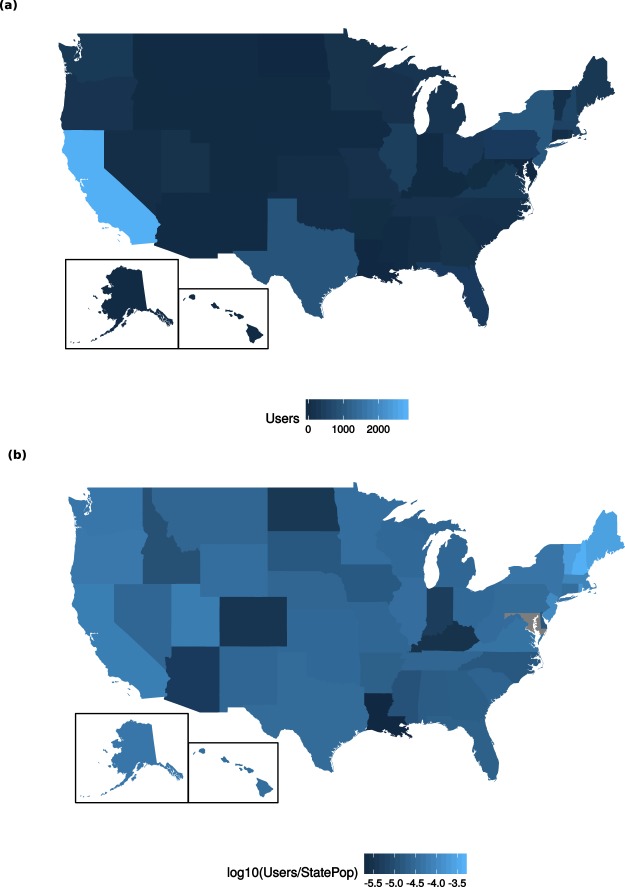
Geographic distribution of 15,578 participants who provided the first three digits of zip codes (self reported) and agreed to broad sharing of information. (**a**) Number of individuals from each state, ranging from n = 0 in Maryland to n = 2,762 in California. (**b**) Number of individuals from each state, normalized by state population as of 2015^27^, ranging from 7.71e-6 in Louisiana to 1.45e-2 in New Hampshire. Values are plotted on a log10 scale. Maryland is grey as no participants were enrolled.

For the 25,774 participants who supplied location data, we illustrate their geographic distribution (https://www.aggdata.com/free/united-states-zip-codes) by state in Fig. 4. The three states with the largest number of participants are California (n = 9,813), New Jersey (n = 3,560), and New York (n = 3,252).

All coded data sets (Table 3) are stored and accessible via the Synapse platform in a public project with associated metadata and documentation^21^.

**Table 3:** Data available for each survey and activity completed by study participants

Survey/Activity Name Data Citation	Description	First Appears	Recurring	#unique users^∗^	#responses
Day One Check-in	Qs about if ready, have device and to get cholesterol reading	Day 1		20,510	22,346
7-Day Activity Assessment https://www.synapse.org/#!Synapse:syn11269541/tables/	Activity state breakdown by timestamp based on core motion phone data	Day 1	Daily	21,382	22,338
Physical Activity Readiness^28^	e4A	Day 1	Every 90 Days	22,136	23,173
Daily Check-in	Qs about wearing wearable, activities (not captured by wearable), sleep	Day 2	Daily	16,593	126,863
Activity and Sleep Survey^29^	e3B, e4A;		Every 90 Days	21,382	22,338
Risk Factor Survey	e5B (Q2-end)	Day 2	Every 90 Days	14,485	15,163
Cardio Diet Survey	e5A	Day 2	Every 90 Days	13,820	14,193
Well-Being and Risk Perception Survey^30^	e4B	Day 2	Every 90 Days	14,168	22,619
Heart/Stroke Risk Score + Heart Age	APHHeartAgeTaskViewController, e5B (Q1)	Day 7	Every 90 Days	4,569	10,366
6-Minute Walk Test		Day 7		3,639	7,317
HealthKit Data https://www.synapse.org/#!Synapse:syn16782062/tables/	Data entries from Health Kit Data types (https://developer.apple.com/documentation/healthkit/health_data_types)	Day 1	Daily	4,920	116,951
HealthKit Sleep https://www.synapse.org/#!Synapse:syn16782061/tables/		Day 1	Daily	626	2,644
HealthKit Workout https://www.synapse.org/#!Synapse:syn16782060/tables/	Data entries from HealthKit Workout Analysis category (https://developer.apple.com/documentation/healthkit/hkworkout)	Day 1	Daily	881	3,267

e3A: Physical Activity Readiness Questionnaire (PAR-Q); e3B: Activity and Sleep Survey: on-the-job activity, leisure-time activity; e4A: Activity and Sleep Survey: Moderate or Vigorous Physical Activity, sleep; e4B: Well-Being and Risk Perception; e5A: Diet Survey; e5B: Cardiovascular Health Survey.

^∗^Users who completed the study more than once from the same email account retained their unique health code identifier for all passes through the study.

## Technical Validation

The survey data provided here are participant reported outcomes responses. For the current data release, participants were allowed to enter survey responses that may not be physiologically possible. This was corrected in version 2 of MyHeart Counts, released Dec 12, 2016 (data not included in this release).

The Core Motion data provided here are derived from Apple iPhone devices with proprietary technical validation. We do not provide test-retest nor other technical validation data sets here, however others have reported technical validation of the Core Motion sensor in a different context^25^.

The 6-Minute Walk Test was validated in an outdoor setting by comparison of step count and distance reported by the *MyHeart Counts* app with corresponding values obtained in accordance with the ATS Statement: Guidelines for the Six-Minute Walk Test (n = 20)^1^. On a validation set of 26 tests, mean error was −3.39 yards, mean absolute error was 56.65 yards, and standard deviation was 70.28 yards^8^. A negative correlation (pearson = −0.58) was found between distance walked and Six-Minute Walk Test error. Due to limitations in how the ActiveTask was encoded, if the study app leaves the foreground during a test, the data collected may not be complete.

### Limitations

The MyHeart Counts study experienced similar limitations as the five other ResearchKit fully-mobile large-scale flagship studies^26^. Although the study recruited over 50,000 users within an interval of 6 months, users did not sustain follow up and there was a significant drop off rate as the mean time of engagement with the app was 4.1 days, consistent with the Asthma Health and mPower studies^22,23^. The high dropout rate was due to low barriers to exit and entry which was a double edge sword as it resulted in both increased dropout but also facilitated engagement of individuals difficult to reach with more traditional means. No sign of systematic bias was found in the characteristics of users who dropped out early versus those who remained in the study longer. Readers may find a more comprehensive list of limitations related to the MyHeart Counts study in a previous publication^8^. Usage Notes

We have instituted governance structures that balance sharing these data for secondary research with commensurate privacy protections for participants:

Researchers interested in accessing these data should complete the following steps:

1. Register for a Synapse account (www.synapse.org)
2. Become a Synapse Certified User by passing a short quiz (www.synapse.org/#!Quiz:Certification)
3. Have their Synapse User Profile validated by the Synapse Access and Compliance Team (ACT)
4. Submit an Intended Data Use statement that is publicly posted
5. Agree to the data-specific Conditions for Use (see DOIs for each data source)

While certain data types may have additional Conditions for Use (e.g., HealthKit data), the overarching Conditions for Use are as follows:

- You confirm that you will not attempt to re-identify research participants for any reason, including for re-identification theory research
- You reaffirm your commitment to the Synapse Awareness and Ethics Pledge
- You agree to abide by the guiding principles for responsible research use and data handling as described in the Synapse Governance documents
- You commit to keeping these data confidential and secure
- You agree to use these data exclusively as described in your submitted Intended Data Use statement
- You understand that these data may not be used for commercial advertisement or to re-contact research participants
- You agree to report any misuse or data release, intentional or inadvertent, to the Access and Compliance Team (ACT) within 5 business days by emailing act@sagebase.org
- You agree to publish findings in open access publications
- You promise to acknowledge the research participants as data contributors and study investigators on all publication or presentation resulting from using these data as follows: ‘These data were contributed by users of the *MyHeart Counts* mobile application as part of the *MyHeart Counts* study developed by Stanford University and described in Synapse^20^.

See the full instructions for requesting data access on the Accessing the *MyHeart Counts* Data page (Wiki, Accessing the *MyHeart Counts* Data^20^).

Examples of client interactions with these data are provided in GitHub: https://github.com/AshleyLab/myheartcounts/tree/DataReleaseManuscript.

## ISA-Tab metadata file

Download metadata file

## Data Availability

The *MyHeart Counts* iOS app (https://github.com/ResearchKit/MyHeartCounts) was built using Apple’s ResearchKit framework (http://researchkit.org/), which is open source and available on GitHub (https://github.com/researchkit/researchkit). It leverages AppCore (https://github.com/ResearchKit/AppCore), a layer built on top of ResearchKit that was shared among the five initial ResearchKit apps. The Bridge iOS SDK (https://github.com/Sage-Bionetworks/Bridge-iOS-SDK) provides integration with Sage Bionetworks’ Bridge Server, a back-end data service designed for collection of participant donated study data (https://developer.sagebridge.org/). The *MyHeart Counts* app can be downloaded on the Apple App Store at (https://itunes.apple.com/us/app/myheart-counts/id972189947?mt=8).
